# Diagnostic Accuracy of Rotational Thromboelastometry for Low-Virulence Periprosthetic Joint Infections: A Pilot Study

**DOI:** 10.3390/microorganisms12081740

**Published:** 2024-08-22

**Authors:** Andreas G. Tsantes, Aglaia Domouchtsidou, Konstantina A. Tsante, Petros Ioannou, Alexandra Mpakosi, Eleni Petrou, Stavros Goumenos, Ioannis G. Trikoupis, Anastasios G. Roustemis, Sotirios P. Fortis, Christos Koutserimpas, Panayiotis J. Papagelopoulos, George Samonis, Stefanos Bonovas, Dimitrios V. Papadopoulos

**Affiliations:** 1 Laboratory of Haematology and Blood Bank Unit, “Attiko” Hospital, School of Medicine, National and Kapodistrian University of Athens, 12462 Athens, Greece; ktsante@yahoo.com (K.A.T.); epetrou@med.uoa.gr (E.P.); 2 Microbiology Department, “Saint Savvas” Oncology Hospital, 11522 Athens, Greece; ldomouchtsidou@gmail.com; 3 Department of Internal Medicine & Infectious Diseases, University General Hospital of Heraklion, 71110 Heraklion, Greece; p.ioannou@uoc.gr; 4 Department of Microbiology, General Hospital of Nikaia “Agios Panteleimon”, 18454 Piraeus, Greece; alexiabakossi@yahoo.gr; 5 First Department of Orthopaedics, School of Medicine, National and Kapodistrian University of Athens, 15771 Athens, Greece; stgoumenos@gmail.com (S.G.); gtrikoupis@hotmail.com (I.G.T.); roustemis.anastasios@gmail.com (A.G.R.); pjporthopedic@gmail.com (P.J.P.); 6 Laboratory of Reliability and Quality Control in Laboratory Hematology (HemQcR), Department of Biomedical Sciences, Section of Medical Laboratories, School of Health & Caring Sciences, University of West Attica (UniWA), 12244 Athens, Greece; sfortis@uniwa.gr; 7 Orthopaedics Surgery and Sports Medicine Department, FIFA Medical Centre of Excellence, Croix-Rousse Hospital, Lyon University Hospital, 69004 Lyon, France; chrisku91@hotmail.com; 8 Metropolitan Hospital, Neon Faliron, 18547 Athens, Greece; samonis@med.uoc.gr; 9 Department of Biomedical Sciences, Humanitas University, 20072 Milan, Italy; sbonovas@gmail.com; 10 IRCCS Humanitas Research Hospital, 20089 Milan, Italy; 11 Second Department of Orthopaedics, School of Medicine, National and Kapodistrian University of Athens, 12462 Athens, Greece; di_papadopoulos@yahoo.gr

**Keywords:** periprosthetic joint infections, low-virulence pathogens, diagnosis, coagulation, rotational thromboelastometry

## Abstract

Background: Periprosthetic joint infections (PJIs) are associated with altered coagulation dynamics; therefore, coagulation laboratory studies could be valuable for diagnosing PJI. This study aimed to evaluate the diagnostic role of Rotational Thromboelastometry (ROTEM) in detecting PJIs caused by low-virulence pathogens. Methods: A retrospective study was conducted, enrolling 78 patients who underwent exchange arthroplasty due to PJI due to high-virulence pathogens (Group A, *n* = 16), low-virulence pathogens (Group B, *n* = 20), or due to aseptic loosening (Group C, *n* = 20). Preoperative laboratory findings were compared among the three groups. Results: Several ROTEM parameters differed in patients with PJIs caused by low-virulence pathogens, indicating a link between these infections and hypercoagulability. The development of low-virulence PJIs was associated with a higher maximum clot firmness (MCF) (Odds Ratio, 1.12; 95% Confidence Interval, 1.04–1.21; *p* = 0.001). Additionally, EXTEM MCF was found to have the highest diagnostic accuracy for these infections (Area Under the Curve, 0.841; sensitivity 90.0%; specificity 90.4%), surpassing that of C-reactive protein and the Erythrocyte Sedimentation Rate (*p* = 0.006 and *p* = 0.019, respectively). Conclusions: Our findings suggest that ROTEM analysis is a promising method for detecting the altered hemostatic dynamics associated with PJI caused by low-virulence pathogens.

## 1. Introduction

The diagnosis of periprosthetic joint infections (PJIs) is challenging since these infections are associated with a vague clinical symptomatology, while the currently available diagnostic tests lack accuracy. Although there are several sets of diagnostic criteria for PJI, there is no single, widely accepted diagnostic strategy for these infections [1]. Currently, the most widely used criteria are based on synovial fluid cultures, inflammatory markers, coagulation-based methods (D-dimers), and histological findings [2,3]. The most essential component of the diagnostic work up is the standard culture of the synovial fluid; however, the sensitivity of standard cultures is low, and in many cases there are false positive results due to contamination. Moreover, although C-reactive protein (CRP) and the erythrocyte sedimentation rate (ESR) are established serum markers due to their low cost and wide availability, these laboratory tests lack specificity; therefore, they cannot be solely used for the diagnosis of periprosthetic joint infections.

The diagnosis of PJI is even more problematic in cases of infections from low-virulence pathogens such as *Staphylococcus epidermidis*, mainly because the sensitivity of synovial fluid cultures for these pathogens is very low, while inflammatory markers are usually within normal values. Although the overall sensitivity of standard synovial cultures for PJI pathogens is estimated to be 72–84%, this rate drops to approximately 40% in the case of low-virulence pathogens [4,5,6]. Newer diagnostic methods such as Next-Generation Sequencing (NGS), which analyzes the DNA sequencing of the collected genetic sample and aligns it with a microbial database, may be promising for the detection of these indolent microorganisms. However, molecular methods such as NGS can result in overdiagnosis, since they are associated with a high rate of false positive results [7,8]. Since the most common pathogens for PJIs include low-virulence pathogens such as coagulase-negative staphylococci, the need for the development of more reliable tests with higher accuracy for PJIs is even more prominent.

The association between altered hemostatic dynamics and infection was established several decades ago. It has been shown that the inflammatory process in infection can be associated with a hypercoagulable state [9,10]. Therefore, coagulation laboratory studies such as viscoelastic studies could be valuable for the detection of PJIs, especially in cases of low-virulence PJIs, for which the currently used methods lack sensitivity and specificity. An example of a viscoelastic method is Rotational Thromboelastometry (ROTEM), which can independently assess different phases of the hemostatic mechanism, providing detailed information regarding any abnormalities in the coagulation cascade. In this context, ROTEM has been evaluated regarding its ability to detect various infections, with promising results [10,11,12]. In a recent study, certain ROTEM parameters were found to have a high diagnostic accuracy for PJIs (sensitivity, 76.6%; specificity, 91.4%), which was comparable with those of CRP and ESR [13].

The purpose of this study was to evaluate the diagnostic role of ROTEM in PJIs caused by low-virulence pathogens. Moreover, we aimed to compare the diagnostic accuracy of ROTEM parameters with that of other conventional tests, such as inflammatory markers and D-dimers.

## 2. Material and Methods

The following study was approved by the institutional review board of the hospital (Ref. number: 199/23-03-2023), and patient confidentiality was appropriately protected.

A pilot retrospective study was conducted, enrolling patients who underwent exchange total hip or total knee arthroplasty due to periprosthetic joint infection or aseptic loosening between October 2021 and June 2024. Patients without laboratory data (including inflammatory markers and ROTEM parameters), patients with coagulopathies, and patients with other infections, such as pneumonia or urinary tract infection, were excluded. The major criteria for the 2018 ICM were used for the definition of PJI (i.e., two positive cultures or presence of a sinus tract), while aseptic loosening was diagnosed when radiographic signs of implant loosening (osteolysis or implant migration) were evident but the criteria for PJI were not met. According to the department’s protocol, all patients undergoing exchange arthroplasty had preoperative joint aspiration, while five tissue samples were obtained in all patients. In all patients with aseptic loosening, the tissue cultures that were obtained during surgery were negative.

In all septic cases, patients were further categorized based on the virulence of the causative pathogens, as either low-virulence PJIs (propionibacterium, coagulase-negative Staphylococci (CNS), including *Staphylococcus epidermidis*) or high-virulence PJIs (*Staphylococcus aureus*, including methicillin resistant *S. aureus* [MRSA], pseudomonas, enterococci, streptococci, candida). Therefore, patients were divided into the following study groups: patients who underwent exchange arthroplasty due to PJI caused by high-virulence pathogens (Group A), patients who underwent exchange arthroplasty due to PJI caused by low-virulence pathogens (Group B), and patients who underwent exchange arthroplasty due to aseptic loosening (Group C).

Patients’ electronic charts were reviewed for the demographics (age, gender, Body Mass Index [BMI], comorbidities), clinical parameters, microbiological results, and preoperative laboratory findings of the included patients. All patients had a preoperative blood work up at the time of their admission to the hospital, one day prior to surgery. This laboratory evaluation included an assessment of inflammatory serum markers (CRP, ESR), standard coagulation tests (prothrombin time [PT], activated partial thromboplastin time [aPTT], D-dimer), and ROTEM analysis.

For the ROTEM analysis, 3–4 mL of a whole blood sample was obtained in a citrated (3.2% sodium citrate) collecting tube. Per ROTEM principles, 300–340 μL of the sample was placed in a disposable cup with the aid of a pipette; after the addition of active reagents, the blood sample was subject to the rotational force of an oscillating cylindrical pin that was immersed in the sample. As the clot was being formed around the pin, the rotation of the oscillating pin was gradually restricted, and this restriction was translated into certain ROTEM parameters. In all cases, the sample was analyzed for its viscoelastic properties within 1.5 h of the blood draw in the ROTEM analyzer (delta ROTEM, Tem Innovation GmbH, Munich, Germany) [14]. The ROTEM analysis included two different assays: the EXTEM assay that evaluates the extrinsic coagulation pathway, and the INTEM assay that evaluates the intrinsic pathway. Different reagents were added to the blood sample for each one of these two assays. Specifically, in order to assess the intrinsic pathway through the INTEM assay, the active reagents that were added to the blood sample included phospholipids, ellagic acid, and Ca2; these reagents activate the intrinsic pathway of the coagulation cascade. Respectively, to assess the extrinsic pathway through the EXTEM assay, tissue factor (TF) was added to the blood sample as an active reagent, activating the extrinsic pathway of the coagulation cascade. The coagulation status of each patient was evaluated through the following ROTEM parameters: the coagulation time (CT, s), reflecting the time taken for a clot that is 2 mm in amplitude to form; the clot formation time (CFT, s), reflecting the time taken for a clot that is 2 mm in amplitude to form after CT; the amplitude of the clot that formed 10 min (A10, mm) after the beginning of the analysis; the alpha angle (a°), which is the angle between the horizontal line (x-axis) and the tangent to the ROTEM trace at a 2 mm clot amplitude; the maximum clot firmness (MCF, mm), which is the maximum clot amplitude; and the lysis index at 60 min (LI60, %), which is calculated as the ratio of the residual clot firmness at 60 min to the MCF.

### Statistical Analysis

This is a pilot study on the diagnostic role of ROTEM analysis for low-virulence PJIs; thus, our study size was not based on a power analysis since a power analysis is not always feasible or necessary for pilot observational studies. The sample size was determined based on practical considerations, including the time available and the number of eligible patients. However, our target was to include a similar or larger population compared to relevant studies that evaluated the association between viscoelastic properties and the development of PJI, such as the one by Yuan et al., which compared the results of thromboelastography (TEG), another viscoelatic method, between patients with and without PJI [15]. Continuous variables are presented as medians and interquartile ranges (IQR), while categorical variables are summarized as frequencies and percentages. The differences between laboratory findings for the three study groups were assessed using the non-parametric Wilcoxon rank-sum (Mann–Whitney) test, while categorical variables were compared using the chi-square test. To evaluate the independent association between infections due to low-virulence pathogens and hemostatic derangements, as reflected by the ROTEM parameters, a multivariable logistic regression analysis was conducted; infection from low-virulence pathogens was the dependent variable, while other parameters such as the ROTEM findings, age and sex were included as independent variables. Additionally, the accuracy of the ROTEM parameters in detecting low-virulence PJIs was evaluated using receiver operating characteristic (ROC) curves, and the areas under these curves (AUC) were calculated. To identify the optimal cut-off value for each parameter, the Youden Index was used, while the respective specificities and sensitivities for these cut-off values were calculated. The Youden Index identifies the cut-off value that maximizes both sensitivity and specificity, by maximizing the difference between the true positive rate and the false positive rate (Youden Index = sensitivity + specificity − 1). Statistical analysis was performed using Stata 18 (Stata Corp., College Station, TX, USA), while a *p*-value of less than 0.05 was considered statistically significant for all tests.

## 3. Results

Overall, 82 patients were evaluated for their eligibility to be included in the study, while four patients with congenital coagulopathy were excluded. The final study population of 78 patients consisted of 16 patients who underwent revision surgery due to high-virulence PJI (Group A), 20 patients who underwent revision surgery due to low-virulence PJI (Group B), and 42 patients who underwent revision surgery due to aseptic loosening (Group C).

The most common isolated pathogens for low-virulence PJIs included Coagulase-negative Staphylococcus species such as *S. epidermidis* (*n* = 9) and *S. lugdunensis* (*n* = 6), while the most common isolated pathogens for high-virulence PJIs included methicillin-resistant *S. aureus* (*n* = 6), methicillin-susceptible *S. aureus* (*n* = 5) and Enterococcus species (*E. faecalis*, *n* = 2; and *E. faecium*, *n* = 1). The three study groups were comparable in terms of age (medians: 69.5 vs. 69 vs. 70 years), gender (males: 50.0% vs. 65.0% vs. 54.7%), smoking status (smokers: 6.2% vs. 10.0% vs. 2.3%), and chronic use of anticoagulants (18.7% vs. 25.0% vs. 30.9%; Table 1). Patients with high-virulence PJIs had a higher BMI compared to patients without PJIs (26.0 vs. 23.0 kg/m^2^; *p* = 0.018).

The findings of conventional coagulation studies including platelets, PT, aPTT and fibrinogen were similar among the three study groups, while the D-dimer levels were higher in patients with high-virulence PJIs compared to those without infections (medians: 1.0 vs. 0.7 mg/L; *p* = 0.013; Table 2). Regarding the inflammatory markers, patients with high-virulence PJIs had higher CRP (medians: 18.0 vs. 3.8 mg/L; *p* < 0.001) and ESR levels (medians: 37.0 vs. 25.0 mm/h; *p* = 0.002) compared to those without infections (Table 2). However, patients with low-virulence PJIs and those without infection had comparable CRP (medians: 12.0 vs. 3.8 mg/L; *p* = 0.16) and ESR levels (medians: 30.5 vs. 25.0 mm/h; *p* = 0.15), indicating that these markers are not suitable for the detection of low-virulence PJIs.

### 3.1. ROTEM Parameters and PJI

**Figure 1:**
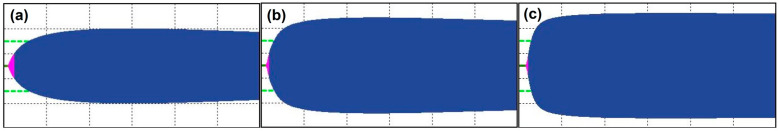
Typical thromboelastograms of a patient with aseptic loosening (**a**), of a patient with a low-virulence periprosthetic joint infection (**b**), and of a patient with a high-virulence periprosthetic joint infection (**c**).

A comparison of the ROTEM parameters revealed that several of them differed between patients with and without PJIs (Figure 1). Specifically, the clot amplitude at 10 min for both the EXTEM and INTEM assay was higher in patients with high-virulence PJIs compared to those without infections (EXTEM A10, medians: 70 vs. 50 mm, *p* < 0.0001; INTEM A10, medians: 79.5 vs. 58 mm, *p* < 0.001), as well as the maximum clot formation for both the EXTEM assay (MCF medians: 81.5 vs. 60.5 mm, *p* < 0.001) and INTEM assay (MCF medians: 82.0 vs. 64.0 mm, *p* < 0.001; Table 3). More interestingly, the same ROTEM parameters differed between patients with low-virulence PJIs and those without infection, indicating that low-virulence infections are associated with altered hemostatic dynamics compared to individuals’ aseptic status, which can be detected by ROTEM analysis. Specifically, the clot amplitude at 10 min for both the EXTEM and INTEM assays was higher in patients with low-virulence PJIs compared to those without infections (EXTEM A10, median: 65 vs. 50 mm, *p* < 0.0001; INTEM A10: 69.5 vs. 58 mm, *p* = 0.001), as well as the maximum clot formation for both the EXTEM assay (MCF medians: 77.0 vs. 60.5 mm, *p* < 0.001) and the INTEM assay (MCF medians: 76.0 vs. 64.0 mm, *p* < 0.001; Table 3).

Multivariable logistic regression analysis further confirmed the association between an increased coagulation dynamic and PJIs caused by low-virulence pathogens (Table 4). Specifically, compared to aseptic status, low-virulence PJIs were associated with a higher EXTEM A10 (Odds Ratio [OR], 1.11, 95% Confidence Interval [CI], 1.04–1.18; *p* = 0.001) and higher INTEM A10 (OR, 1.09, 95% CI, 1.01–1.17; *p* = 0.015). Similarly, low-virulence PJIs were associated with a higher EXTEM MCF (OR, 1.12, 95% CI, 1.04–1.18; *p* = 0.001) and higher INTEM MCF (OR, 1.14, 95% CI, 1.04–1.25; *p* = 0.003) compared to aseptic status.

### 3.2. Diagnostic Accuracy of ROTEM Parameters

The ROTEM parameters demonstrated a good capacity to discriminate PJIs (caused by both low- and high-virulence pathogens) from aseptic cases based on the AUC values. Specifically, the AUC value of EXTEM A10 for PJI was 0.825 (95% CI, 0.712–0.939), the AUC value of EXTEM MCF was 0.879 (95% CI, 0.788–0.970), the AUC value of INTEM A10 was 0.790 (95% CI, 0.671–0.909) and the AUC value of INTEM MCF was 0.829 (95% CI, 0.722–0.936).

**Figure 2:**
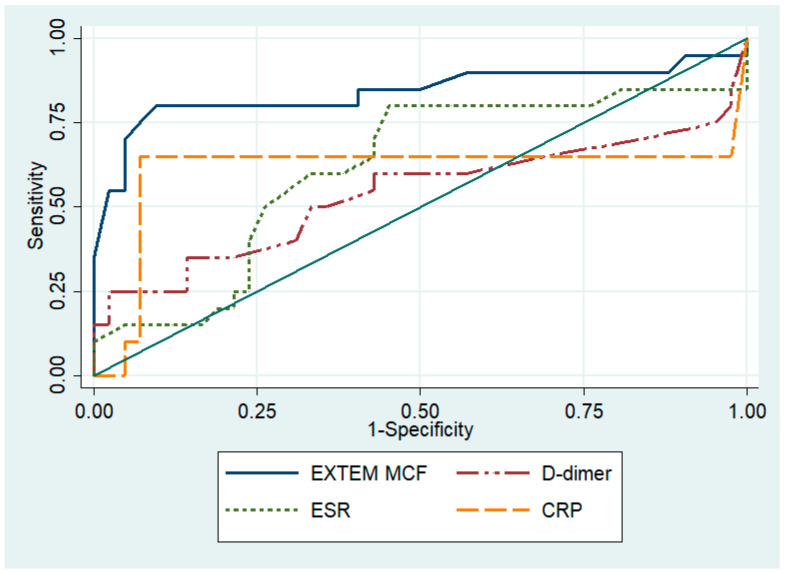
The areas under the receiver operating characteristic curve for the D-dimer levels, erythrocyte sedimentation rate (ESR), C-reactive protein (CRP), and EXTEM MCF for the diagnosis of periprosthetic joint infections caused by low-virulence pathogens.

Regarding low-virulence PJIs, the highest diagnostic accuracy was found for EXTEM MCF (AUC, 0.841; 95% CI, 0.704–0.978), followed by INTEM MCF (AUC, 0.826; 95% CI, 0.687–0.964; Table 5). A cut-off value of ≥70 mm for EXTEM MCF was revealed to have 80.0% sensitivity and 90.4% specificity to identify an altered homeostasis status that is associated with low-virulence PJIs. Regarding the diagnostic accuracy of inflammatory markers for low-virulence PJIs, CRP (AUC, 0.610; 95% CI, 0.406–0.813) and ESR (AUC, 0.613; 95% CI, 0.451–0.775) demonstrated low performance (Figure 2).

EXTEM MCF and INTEM MCF had similar diagnostic accuracies (*p* = 0.80). However, EXTEM MCF was found to have higher diagnostic accuracy than CRP (*p* = 0.006) and ESR (*p* = 0.019), and a higher diagnostic accuracy than D-dimer (*p* = 0.002).

## 4. Discussion

The diagnosis of PJI mainly relies on standard cultures that are prone to false negative results. The detection of PJI caused by low-virulence pathogens is even more challenging, since the sensitivity of standard cultures for these pathogens is even lower, while inflammatory markers are usually within the normal range. Therefore, new diagnostic strategies should be explored. In this direction, the association between infection and altered hemostatic dynamics may provide a promising route for the development of novel diagnostic tests with a higher accuracy. Rotational thromboelastometry is a coagulation-based method that has been shown to have good diagnostic capabilities for infections [13,15]. Based on the findings of this study, ROTEM analysis may be also a suitable test for the detection of PJIs caused by low-virulence pathogens. Specifically, we found that EXTEM MCF had a high diagnostic accuracy for low-virulence PJIs (AUC, 0.841; 95% CI, 0.704–0.978), comparable to that of INTEM MCF (AUC, 0.826; 95% CI, 0.687–0.964). Notably, EXTEM MCF was found to have a higher diagnostic accuracy than CRP (*p* = 0.006) and ESR (*p* = 0.019), and a higher diagnostic accuracy than D-dimer (*p* = 0.002) for these infections. Moreover, we found that a cut-off value ≥ 70 mm for EXTEM MCF is associated with 80.0% sensitivity and 90.4% specificity for the identification of an altered hemostatic status that is related to low-virulence PJIs.

Several studies have shown that there is a positive relation between inflammation and hypercoagulability [10,16,17]. Since infections are associated with several inflammatory pathways, the association between inflammation and hypercoagulability can be easily applied to the positive relation between infections and hypercoagulability. The hypercoagulability in patients with infections has been attributed to several mechanisms such as the amplification of the extrinsic pathway of the coagulation cascade, cytokine-induced coagulation enhancement, and hypofibrinolysis. Saxena et al. evaluated the hemostatic abnormalities in patients with PJIs and reported that the overexpression of factor VIIa can be attributed to the release of various inflammatory cytokines, while direct endothelial injury caused by infective pathogens can also result in the amplification of the coagulation cascade [18]. The results of our study support the positive relationship between infection and hypercoagulability, since the increased formation of thrombin, as indicated by the increased maximum clot firmness, was strongly related to PJI.

Inflammatory markers such CRP and ESR remain essential components of the diagnostic work up for PJI. In daily clinical practice, ESR has been widely replaced by CRP since ESR is affected by several parameters such as anemia and protein levels; meanwhile, ESR measurements remain elevated for a longer period [19]. However, CRP may not be suitable for the diagnosis of low-virulence pathogens, since in many cases these infections are associated with low CRP levels [20,21,22]. Notably, the estimated rate of normal CRP values in low-virulence PJIs has been reported to be up to 70% [23]. This was also evident in a recent study in which the diagnostic accuracy of D-dimer for PJIs was compared to that of inflammatory markers in 412 patients [9]. The authors of this study reported that although the overall diagnostic accuracy of D-dimer was similar to that of ESR and CRP, D-dimer had a higher sensitivity than CRP and ESR in infections resulting from low-virulence pathogens. The results of this study further support that coagulation-based studies may be more suitable than CRP and ESR for the detection of PJIs caused by low-virulence pathogens. Interestingly, our results indicate that ROTEM parameters are more sensitive coagulation biomarkers than D-dimer for low-virulence PJIs; therefore, ROTEM parameters may be more even valuable than D-dimer for the detection of PJIs caused by indolent pathogens.

Although there are two studies evaluating the diagnostic capacity of viscoelastic studies for PJIs, the association between the virulent behavior of PJI pathogens and the diagnostic accuracy of these studies was not evaluated in any of them. In the first one, the results of several laboratory tests, including thromboelastography (TEG), were compared between patients who underwent exchange arthroplasty due to aseptic loosening or PJI [15]. The highest diagnostic accuracy was revealed for ESR, while a TEG parameter called maximum amplitude (MA), which is similar to the MCF parameter in ROTEM analysis, was found to have the second highest accuracy, followed by CRP. However, a direct comparison of the diagnostic values of these parameters was not performed in this study. The maximum amplitude in TEG analysis reflects thrombin generation, similar to MCF, which was found to have the highest diagnostic accuracy in our study. In the second study evaluating the role of viscoelastic studies in PJIs, the ROTEM results were compared between 30 patients who underwent exchange arthroplasty due to PJI and 35 patients who underwent exchange arthroplasty for aseptic loosening [13]. The ability of EXTEM MCF to identify patients with PJI was similar to that of serum markers such as CRP and ESR. The results of both these studies highlight the association between PJIs and hypercoagulability caused by increased thrombin generation. Notably, the results of the current study indicate that this association may be even more valuable in infections caused by low-virulence pathogens and should be explored as a diagnostic strategy for those difficult cases.

Interestingly, several ROTEM parameters such as EXTEM MCF were outside of the normal reference range in patients with PJI, indicating a hypercoagulable profile. Although all patients undergoing revision arthroplasty receive thromboprophylaxis postoperatively, the hypercoagulable profile that was revealed in PJI patients indicates that an enhanced thromboprophylaxis regime may be advisable in these patients. However, there is a lack of evidence regarding the association between the development of postoperative VTE and preoperative ROTEM values in patients undergoing revision hip or knee arthroplasty; therefore, recommendations regarding the thromboprophylactic protocol cannot be made based on ROTEM values. Future studies should investigate whether this hypercoagulable profile based on ROTEM parameters is associated with an increased rate of thromboembolic events, and whether modification in thromboprophylaxis based on ROTEM values is advisable.

In recent years, several studies have investigated novel diagnostic strategies for the identification of low-virulence PJI pathogens. In this context, molecular diagnostic methods such as polymerase chain reaction (PCR) and NGS have emerged [24]. NGS analyzes the DNA sequencing of the collected genetic sample and aligns it with a microbial database. These methods have the advantage of needing less time to identify the causative pathogen, while previous antibiotic therapy does not have any impact on the identification process. In a recent study, the sensitivity and specificity of multiplex PCR in the detection of PJI pathogens were evaluated [24]. The authors reported that molecular testing had yielded a sensitivity of 80% and sensitivity of 100% for PJIs. However, the authors highlighted that there were some discrepancies between the results of standard cultures and PCR, with PCR more often identifying indolent pathogens. However, the results of NGS should be interpreted with caution in light of recent findings in the field of musculoskeletal infections indicating that synovial fluid is not aseptic. DNA sequencing technology enabled researchers to detect pathogens in the synovial fluid of healthy individuals, indicating that certain pathogens can be considered as part of the so-called normal synovial biome [25]. Therefore, the clinical importance of a positive finding in NGS is debatable, since it can reflect a normal finding and not an infection. In other words, NGS is associated with a high rate of false positive results; thus, further research is needed in order to set a more robust definition and understanding of what a positive NGS finding reflects regarding its association with a clinically evident PJI.

There are certain limitations in this study that need to be addressed. The small population of this study, especially regarding the subgroup of patients with low-virulence PJIs poses a certain limitation; therefore, the results of this study should be interpreted with caution. However, this is a pilot study evaluating the diagnostic accuracy of ROTEM analysis for these infections without any similar studies in the literature. Future studies enrolling larger populations should be conducted in order to validate our findings. Second, several conditions, such as venous thromboembolism, can influence the results of coagulation-based studies such as ROTEM analysis and D-dimer. The clinical signs of thrombosis were not evident in any of our patients at the time of evaluation prior to exchange arthroplasty, while one patient in the aseptic loosening group developed deep vein thrombosis postoperatively. However, we have to note that routine lower extremity Doppler in order to rule out subclinical thrombosis was not performed. Third, the study was designed to include patients with PJIs based only on the major criteria of the 2018 ICM; therefore, our results cannot be applied to patients who are meeting the minor criteria for PJI. This was done because laboratory parameters such as the CRP, ESR and D-dimer level are included in the minor criteria of 2018 ICM. Therefore, since the diagnostic accuracy of these parameters is evaluated in this study, a definition of PJI based on these criteria would result in biased outcomes.

The currently available sets of diagnostic tests are associated with a low sensitivity to PJIs caused by low-virulence pathogens. These pathogens are difficult to isolate in standard synovial fluid cultures, and are associated with low CRP and ESR levels. Novel diagnostic modalities such as NGS are promising; however, there are certain issues such as the high false positive rate that need further investigation. Moreover, these studies are associated with a high cost. In this context, the association between infection and altered hemostatic dynamics may be valuable in order to develop accurate diagnostic tests for these infections. Our results indicate that ROTEM analysis is a promising method for the detection of the altered hemostatic dynamic that is associated with the development of PJI due to low-virulence pathogens. The value of incorporating viscoelastic studies into diagnostic strategies for periprosthetic joint infections caused by low-virulence pathogens should be investigated in further multicenter studies with larger populations.

## Figures and Tables

**Table 1:** Demographics and clinical characteristics of the study population.

Variables	High-Virulence PJI (Group A, *n* = 16)	Low-Virulence PJI (Group B, *n* = 20)	Aseptic Loosening (Group C, *n* = 42)	*p*-Values		
				A vs. B	A vs. C	B vs. C
Age (years)	69.5 (65.5–73)	69 (66.5–71)	70 (67–72)	0.72	0.89	0.47
Gender (males, %)	8 (50.0)	13 (65.0)	23 (54.7)	0.50	0.77	0.58
BMI (kg/m^2^)	26 (24–29)	25.5 (22.5–26.5)	23 (21–25)	0.19	0.018	0.059
CCI	5 (3.5–5)	4 (3–5.5)	4 (3–5)	0.19	0.06	0.85
Smoking	1 (6.2)	2 (10.0)	1 (2.3)	0.99	0.47	0.24
Operated joint (THA)	11 (68.7)	14 (70.0)	27 (64.2)	0.99	0.99	0.99
Anticoagulants	3 (18.7)	5 (25.0)	13 (30.9)	0.70	0.51	0.76
Antiplatelets	1 (6.2)	3 (15.0)	6 (14.3)			
VKA	0 (3.3)	0 (0.0)	1 (2.3)			
NOACs	2 (12.5)	2 (10.0)	6 (14.3)			

Footnotes: Data are presented as medians and interquartile ranges (IQR), or as frequencies and percentages when appropriate. The non-parametric Wilcoxon rank-sum test and the chi-square test were used for the comparison between groups. Abbreviations: BMI, Body Mass Index; CCI, Charlson Comorbidity Index; PJI, periprosthetic joint infections; THA, total hip arthroplasty; VKA, vitamin K antagonists; NOAC, novel oral anticoagulant.

**Table 2:** Conventional laboratory assays of the study cohort.

Variables	High-Virulence PJI (Group A, *n* = 16)	Low-Virulence PJI (Group B, *n* = 20)	Aseptic Loosening (Group C, *n* = 42)	*p*-Values		
				A vs. B	A vs. C	B vs. C
PLTs (×10^3^/mL)	284.0 (249.0–303.5)	268.5 (249.0–289.5)	261.0 (216.0–300.0)	0.47	0.34	0.65
aPTT (sec)	30.4 (29.3–34.2)	32.0 (29.8–34.2)	32.0 (30.0–34.0)	0.66	0.64	0.86
PT (sec)	12.1 (11.7–12.8)	12.4 (11.9–14.5)	12.0 (11.0–14.0)	0.37	0.86	0.46
D-dimers (mg/L)	1.0 (0.7–2.4)	0.85 (0.35–1.35)	0.7 (0.5–1.0)	0.21	0.013	0.58
Fibrinogen (mg/dL)	428.0 (360.0–470.0)	410.0 (385.0–457.5)	398.0 (360.0–456.0)	0.69	0.26	0.31
CRP (mg/L)	18.0 (8.5–24.0)	12.0 (0.1–15.0)	3.8 (3.4–4.1)	0.006	<0.001	0.16
ESR (mm/h)	37.0 (34.0–39.5)	30.5 (26.0–34.0)	25.0 (19.0–31.0)	0.011	0.002	0.15

Footnotes: Data are presented as medians and interquartile ranges (IQRs). The non-parametric Wilcoxon rank-sum test was used for the comparison between groups. Abbreviations: PJI; periprosthetic joint infections; PLTs, platelets; aPTT, activated partial thromboplastin time; PT, prothrombin time; CRP, C-reactive protein; ESR, erythrocyte sedinentation rate.

**Table 3:** ROTEM parameters of patients with and without PJI infection.

Variables	High-Virulence PJI (Group A, *n* = 16)	Low-Virulence PJI (Group B, *n* = 20)	Aseptic Loosening (Group C, *n* = 42)	*p*-Values		
				A vs. B	A vs. C	B vs. C
EXTEM CT (s)	64.5 (57.0–75.5)	68.0 (59.5–86.5)	65.0 (61.0–69.0)	0.21	0.89	0.14
EXTEM CFT (s)	54.5 (49.0–76.0)	60.5 (50.5–90.0)	85.0 (78.0–93.0)	0.33	<0.001	0.010
EXTEM A10 (mm)	70.0 (62.5–75.0)	65.0 (57.5–69.5)	50.0 (46.0–53.0)	0.14	<0.001	<0.001
EXTEM MCF (mm)	81.5 (76.0–84.0)	77.0 (70.5–79.0)	60.5 (56.0–66.0)	0.035	<0.001	<0.001
EXTEM Alpha angle	70.0 (67.5–75.5)	71.5 (68.0–75.0)	72.5 (68.0–75.0)	0.71	0.70	0.88
EXTEM LI60 (%)	93.5 (91.5–96.5)	93.0 (89.5–95.0)	91.5 (89.0–95.0)	0.19	0.62	0.58
INTEM CT (s)	179.0 (146.0–204.5)	187.0 (154.4–227.5)	180.0 (175.0–186.0)	0.44	0.57	0.50
INTEM CFT (s)	48.0 (39.0–69.5)	59.5 (48.0–91.5)	70.0 (66.0–77.0)	0.12	0.003	0.42
INTEM A10 (mm)	79.5 (68.5–82.5)	69.5 (60.5–77.0)	58.0 (56.0–63.0)	0.029	<0.001	0.001
INTEM MCF (mm)	82.0 (72.5–84.0)	76.0 (68.0–81.5)	64.0 (61.0–68.0)	0.21	<0.001	<0.001
INTEM Alpha angle	77.5 (79.0–80.0)	77.0 (69.0–80.0)	75.0 (69.0–78.0)	0.94	0.50	0.48
INTEM LI60 (%)	95.5 (92.0–97.5)	92.5 (91.0–94.5)	92.0 (87.0–94.0)	0.035	0.004	0.34

Footnotes: Data are presented as medians and interquartile ranges (IQRs). The non-parametric Wilcoxon rank-sum test was used for the comparisons between groups. Abbreviations: PJI; periprosthetic joint infections; CT, clotting time; CFT, clot formation time; A10, clot amplitude at 10 min; MCF, maximum clot firmness; LI60, lysis index at 60 min.

**Table 4:** Results of multivariable logistic regression analysis for periprosthetic joint infection due to low-virulence pathogens (dependent variable), with ROTEM parameters, age, sex, BMI, Charlson comorbidity index, smoking status and chronic anticoagulants included in the models as independent variables.

Variables	Low-Virulence Periprosthetic Joint Infection		
	OR	(95% CI)	*p*-Value
EXTEM CFT	0.99	(0.97–1.01)	0.60
EXTEM A10	1.11	(1.04–1.18)	0.001
EXTEM MCF	1.12	(1.04–1.21)	0.001
EXTEM LI60	1.05	(0.91–1.22)	0.44
INTEM CFT	1.00	(0.99–1.02)	0.30
INTEM A10	1.09	(1.01–1.17)	0.015
INTEM MCF	1.14	(1.04–1.25)	0.003
INTEM LI60	1.12	(0.97–1.31)	0.11
CRP	1.00	(0.95–1.05)	0.97
ESR	1.02	(0.97–1.07)	0.40
D-dimer	1.59	(0.62–4.07)	0.62

Abbreviations: OR, odds ratio; CI, confidence interval; CFT, clot formation time; A10, clot amplitude at 10 min; MCF, maximum clot firmness; LI60, lysis index at 60 min; CRP, C-reactive protein; ESR, erythrocyte sedimentation rate.

**Table 5:** Accuracy of ROTEM parameters and conventional studies for periprosthetic joint infection due to low-virulence pathogens.

Parameter	Periprosthetic Joint Infection				
	AUC (95% CI)	Optimal Cut-Off	OR (95% CI)	Sensitivity (%)	Specificity (%)
EXTEM A10	0.797 (0.633–0.960)	≥56	24.0 (5.9–96.8)	80.0	85.7
EXTEM MCF	0.841 (0.704–0.978)	≥70	37.9 (8.4–170.0)	80.0	90.4
INTEM A10	0.747 (0.580–0.914)	≥66	11.6 (3.4–40.8)	70.0	83.3
INTEM MCF	0.826 (0.687–0.964)	≥75	17.2 (4.5–65.7)	60.0	100.0
Fibrinogen	0.579 (0.428–0.731)	≥406	2.2 (0.7–6.5)	60.0	59.5
D-dimer	0.542 (0.360–0.725)	≥1.5	6.6 (1.1–38.1)	25.0	97.6
CRP	0.610 (0.406–0.813)	≥6	24.1 (5.4–107.2)	65.0	92.8
ESR	0.613 (0.451–0.775)	≥26	4.8 (1.3–16.9)	80.0	54.7

Abbreviations: AUC, area under curve; OR, odds ratio; CI, confidence interval; A10, clot amplitude at 10 min; MCF, maximum clot firmness; CRP, C-reactive protein; ESR, erythrocyte sedimentation rate.

## Data Availability

The raw data supporting the conclusions of this article will be made available by the authors upon request.
